# The Treatment of Tabes Dorsalis

**Published:** 1899-12-16

**Authors:** 


### THE TREATMENT OF TABES DORSALIS.

In a paper read before the New York Academy of Medicine Dr. Charles L. Dana[1] gives a good outline of the treatment which it is well to adopt in cases of tabes dorsalis. In the first and second stages all cases with a distinct history of primary or secondary syphilis, either cutaneous or in the nerve centres, should receive rigorous courses of mercury and iodide. Such patients are in the minority, but when met with they should receive the treatment. They should be kept at home, if possible, and in bed. The best way to give mercury is by hypodermic injections of corrosive sublimate, in doses of grain 1-20th once a day, or twice if the symptoms are urgent. They should be given for two or four weeks according as constitutional symptoms appear, the patient taking about 30 grains of iodide of potassium daily during this period; and after the mercurial course the iodide is to be rapidly increased, 300 grains a day being given if possible. This medication is continued for about four weeks, so that the patient is under treatment for six to eight weeks. He should have during all this time a warm bath twice a week, and oftener if he desires it. If the hypodermics are not well borne the patient should receive inunctions of mercurial ointment for six successive days, and a hot bath on the seventh, this being repeated for three or four weeks. After this time the patient receives tonic treatment—iron and some preparation of phosphorus, such as phosphoric acid or glycero-phosphate of lime or soda. Such measures may be continued for two months, during which time, also, he receives luke-warm baths, followed sometimes by cool affusion, or he may be sent to some thermal springs. During the tonic period counter irritation to the spine may be made by the cautery or small blisters, the former being applied twice a week if there is much pain. Patients should rest as much as possible during all this time, which covers about three months. Driving is good as a form of exercise, walking is bad. The patient may smoke and take a moderate amount of alcohol, and should be allowed to look after his affairs and take his share in the enjoyments of life. The anti-syphilitic and bath treatment should be repeated at least once a year, and in the interval the patient is given tonic medication and mechanical treatment of various kinds.

Supposing, however, that the patient has no history either of syphilis or its sequelæ, it is useless to put him through the mercury and iodide treatment, and it is not quite certain that such treatment may not do him harm. These purely degenerative cases must be treated at once as if they were getting prematurely old, as, in fact, they are, *quoad* the spinal sensory neuron. They must be put at once on the two months' rest, tonic, and bath regimen. Then the question arises whether we have any specific measure by which the progressive decay can be stopped or slowed up. Such a specific is to some extent constituted by a regular, quiet institution life, such as is found in hospitals, and such as cannot always be easily imitated at home. In this way the poor man who comes into hospital is often more surely helped than the rich man, who has the luxuries and anxieties of a home and family. But there is one method which is on the whole more successful than any other single proceeding, and it may be looked upon also as measurably specific. It consists in putting the patient to bed and giving him large doses of strychnine and small doses of morphine by hypodermic injection. Phosphates and iron may be given at the same time. But strychnine, although a useful drug in tabes, is also apt to be a dangerous one, for it may produce pains and even a kind of general collapse of the patient, as though the motor cells were exhausted. Guarded by rest and small amounts of morphine, however, Dr. Dana has never seen any harm result, and almost always much benefit. So far as bathing is useful, it can often be done at home as well as at the spring. Some patients are distinctly improved by careful courses of electrical treatment, including galvanisation of the spine and general faradisation of the muscles, while other persons are annoyed and irritated by it. No doubt the effect of such treatment is partly psychical. Muscle training, according to the system of Dr. Fraenkel, so as to re-educate the muscular sense, is often useful. It is adapted especially to cases in the first or second stage which have ceased to progress, and in which there is some muscular power. Sometimes, even in cases which are bedridden in the third stage of the disease, but in which the malady is practically arrested, this method is of service. But in this stage treatment has to be principally directed to the relief of pain and visceral troubles.

[1] Med. Rec., Nov. 18.

